# *Aerococcus urinae* Mitral Valve Endocarditis-Related Stroke: A Case Report and Literature Review

**DOI:** 10.1177/2324709618758351

**Published:** 2018-02-25

**Authors:** Darius Adomavicius, Mark Bock, Christian-Friedrich Vahl, Ekkehard Siegel

**Affiliations:** 1 University Medical Centre Mainz, Mainz, Germany; 2 Helios Dr Horst Schmidt Clinics Wiesbaden, Wiesbaden, Germany

**Keywords:** *Aerococcus urinae*, infective endocarditis, heart valve, stroke, emboli

## Abstract

*Background. Aerococcus urinae* is a rare causative pathogen of infective endocarditis that results in a high risk of embolic events. The mortality rate for *A urinae* endocarditis is high. Old age and underlying urologic conditions are the best-known risk factors for infection. *Case Description.* We report the clinical course of the disease in a 49-year-old man who presented symptoms of a urinary tract infection. A few days later, transthoracic echocardiography showed a conspicuous mitral valve with myxomatous alterations. Following the detection of a cerebral embolism with associated stroke symptoms, as well as at the beginning of cardiac failure, the emergency indication for the surgical treatment of mitral valve endocarditis was given. On the second day following the operation, circulatory collapse rapidly developed. Following an unsuccessful attempt at cardiopulmonary resuscitation, the patient died. *Review of the Literature.* From 1991 to 2017, 29 cases of *A urinae*–induced endocarditis have been described in PubMed and Medline. One or 2 new cases are published annually. We review all reported cases of *A urinae* endocarditis, with an emphasis on the predisposing factors, course, and outcomes of the disease. *Conclusion. A urinae* endocarditis is a rare disease primarily affecting elderly men with urinary tract pathologies and comorbidities. The course of the disease is severe, and the outcome is often fatal. A 16S rDNA polymerase chain reaction investigation of bacterial genome provides proof of the presence of *A urinae*. Because of the high risk of embolism, rapid treatment should focus on the diseased heart valve. Based on existing data and the experience gained from handling cases, treatment with β-lactam and aminoglycosides is recommended. It is also recommended that operative therapy take place as soon as possible.

## Introduction

*Aerococcus urinae* is a gram-positive bacterium, a catalase-negative coccus. It was first described in the literature in the 1990s as a bacterium that causes urinary tract infections.^1^ The organism is considered to be of low pathogenicity, and treatment may not always be required.^2,3^ *A urinae* has been shown to cause invasive infections such as sepsis, urinary tract infection, and infective endocarditis (IE) in humans.^4,5^ The mortality rate from *A urinae* endocarditis is high. Furthermore, *A urinae* can form biofilms on foreign materials and aggregate human platelets, which makes it potentially virulent.^4,6^ Old age and underlying urologic conditions are the best-known risk factors for infection.^2,7,8^

The incidence of *A urinae* urinary tract and bloodstream infections is estimated to be 54 and 3 per 1 000 000, respectively, per year.^8^

## Microbiology

**Figure 1.**
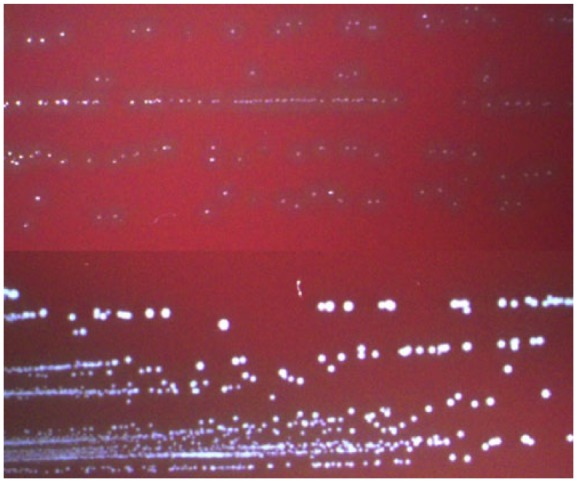
The upper part of the figure shows a blood isolate of *Aerococcus urinae* grown in blood agar with 5% CO_2_ for 24 hours. Small colonies with α-hemolysis can be seen clearly. The lower part of the figure shows a blood isolate of *Aerococcus sanguinicola* grown on the same plate. The colonies are larger, whiter, and the hemolysis is not as pronounced.^3^

*Aerococcus* species appear in clusters (see Figure 1) as gram-positive cocci. However, in contrast to *staphylococci*, they are catalase negative.^9^ Testing for leucine aminopeptidase, which is positive only for *A urinae*, can be used to differentiate the latter.^10,11^ Furthermore, the Vitek system is a reliable instrument for phenotypical identification, and 16S rDNA polymerase chain reaction (PCR) may be used for genotypical confirmation. In cases of negative blood cultures, the investigation and determination of 16S rDNA PCR directly in a blood culture may reveal *A urinae*.^12^ Moreover, it is possible to determine 16S rDNA in the heart valve after valve-replacement surgery. The results obtained from these methods directly confirm the diagnosis of *A urinae* endocarditis.

## Case Presentation

The 49-year-old German male was admitted to the hospital by his general practitioner. He had Down syndrome (Trisomy 21) and was supported by his parents at home. The patient was suffering from a urinary tract infection that had been pretreated with ciprofloxacin under outpatient conditions. In the course of the oral ciprofloxacin treatment, the patient experienced an undulating fever as well as a recurrence of the infecting parameters.

The patient was with an altered mental status, awake but not oriented to his surroundings nor responsive to attempts to communicate with him. There were no heart murmurs to be auscultated. The heart action was rhythmic though mildly tachycardic. An auscultation of the lungs presented vesicular breath sounds, and an inspiratory and expiratory hum. Sonorous percussion was evident. The abdomen was soft, and the bowel sounds were properly distributed in all quadrants. Kidney percussion was not painful, and there were no signs of peripheral edema or venous congestion on the neck.

His electrocardiogram on the day of hospitalization was normal, with regular rhythm at 82 beats per minute.

A chest X-ray (posteroanterior view) showed a normal heart, no reliable detection of infiltration, and small pleural effusion though with an emphysema-like appearance.

**Figure 2.**
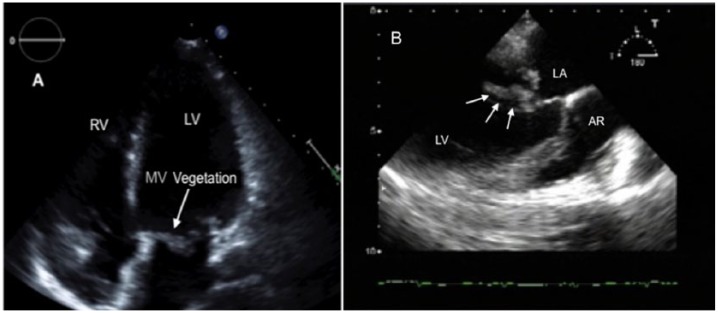
Transthoracic echocardiogram (A) showing a considerable vegetation on the MV. Transesophageal echocardiographic image (B) displays native MV endocarditis. The white arrows show a mobile 23-mm echodense structure on the anterior MV leaflet. MV, mitral valve; RV, right ventricle; LV, left ventricle; LA, left atrium; AR, aortic root.

Transthoracic echocardiography on seventh day showed a conspicuous mitral valve with myxomatous alterations (see Figure 2A), though with otherwise normal findings.

Transesophageal echocardiography on the same day gave the results described below. The mitral valve had vegetation located on the anterior leaflet, segment 3, with about 23 × 11 mm oscillating structure on it (see Figure 2B). In the same area, there was damage to the valve with a difficult-to-quantify eccentricity with an at least moderate degree of insufficiency. The other flaps were morphologically and functionally inconspicuous. There was good contractile left ventricle. Left atrial appendage was free from thrombi with good flow.

An abdominal ultrasound exam showed the massively distended bladder, and the other abdominal structures were observed and were normal. Because of the urinary obstruction and phimosis, a suprapubic catheter was placed. Urine sample was taken to microbiological investigation. There was no growth of bacteria.

On the eighth day of hospitalization, the blood cultures were obtained. After 50 hours of cultivation, *A urinae* was detected in aerobic cultures. The blood was taken during treatment with ciprofloxacin. A resistance profile showed sensitivity to penicillin, ampicillin, meropenem, and resistance to ciprofloxacin and levofloxacin.

After changing the antibiotic to intravenous gentamicin and ceftriaxone, there was a decrease of leukocytes and C-reactive protein so thus a slight clinical improvement in the general condition of the patient.

On the 11th day, the patient was transferred to a cardiac center for further, possibly surgical, treatment of mitral valve endocarditis. A transthoracic echocardiogram taken on the same day showed normal functioning of the left ventricle with an ejection fraction of over 55%, and normally sized left and right ventricle without hypertrophy. The mitral valve had strong myxomatous alterations of the anterior leaflet with a clear prolapse on it, and high mitral regurgitation. No relevant pericardial effusion could be detected.

To clarify the mitral valve regurgitation, a transesophageal echocardiography was carried out. This also indicated vegetation on the mitral valve.

## Laboratory Findings

As may be expected from a bacterial infection, levels of leukocytes and C-reactive protein had increased—although the subsequent increase of both was partly associated with the surgery. In addition, creatinine and urea increased in the course of treatment, which indicated acute renal failure (see Table 1). Furthermore, cardiac enzymes developed during the course of treatment (see Table 2). An acute myocardial infarction as a result of ischemic embolic events could not be excluded. The other laboratory findings such as those concerning liver parameters and chemistry panel including coagulation parameters showed no clinically relevant change.

**Table 1.** Laboratory Findings. On the 13th Day, the Patient Had an Operation.

Analyte, Reference	Day 1	Day 4	Day 6	Day 8	Day 13	Day 14	Day 15
C-reactive protein, <5 mg/L	113.3	67.5	93.1	130.3	82	67	126
Leukocytes, 3.5-10/nL	7.6	8.2	5.7	6.6	20.9	26.4	24.6
Creatinine, 0.7-1.3 mg/dL	1.2	1.0	1.2	1.0	2.4	2.7	3.2
Urea, 9-21 mg/dL	45	30	36	36	28	51	63

**Table 2.** Laboratory Findings. On the 13th Day the Patient Had an Operation.

Analyte, Reference	Day 1	Day 13	Day 14	Day 15
Troponin I, <24 pg/mL	—	—	81 543	91 995
Creatinine kinase, 30-200 U/L	409	150	1350	1176
Creatinine kinase-MB, <25 U/L	10.7	—	273	236

**Figure 3.**
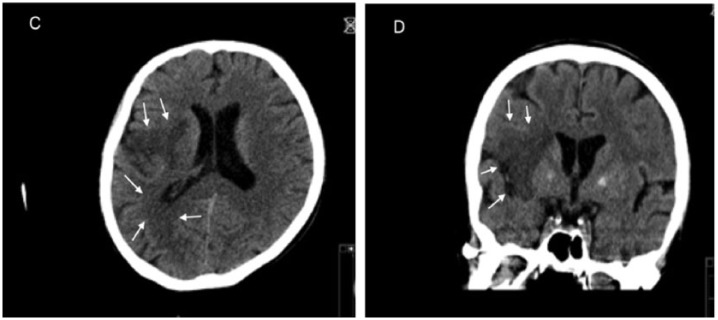
Computed tomography of the head—transverse plane (C) and frontal plane (D)—displaying hypodense areas (white arrows) consistent with subacute cerebral infarctions.

Computed tomography of the head, on the 13th day, showed a subacute middle cerebral artery infarct on the right side without significant hemorrhagic transformation (see Figure 3C and D). Following the detection of a cerebral embolism with associated stroke symptoms, as well as at the beginning of cardiac failure with tachycardia, tachypnea, and persistent fever, an emergency indication was given for the operative treatment of the mitral valve.

## Operational Approach and Findings

The operation was performed through a median sternotomy with cardiopulmonary bypass. Intraoperative findings showed chordae tendineae rupture of the posteromedial papillary muscle with a calcified ring in the region P3/A3, as well as vegetation on the ring and on the papillary muscle itself. The anterior mitral valve leaflet and tendon cord, as well as the P3 segment were sent as a sample to the pathology department and the abraded vegetation to the microbiology department. The P1/P3 segments were not removed. A Hancock II 29-mm BioClave was subsequently inserted into the mitral position.

## Microbiological Findings and Genome Detection of *A urinae*

Microscopic examination of the preparation revealed the findings that follow. The valve stroma was slightly fibrosized with thrombotic attachments. Inflammatory cells, in the form of granulocytes and histiocytes, had increased in number. The inflammatory reaction was spread over the whole valve stroma. In summary, there is no doubt that in the present sample of the mitral valve, endocarditis ulceropolyposa (infectious endocarditis) is present. During the microbiological examination, the material examined was free from bacteria. In the course of the 16S rDNA PCR investigation of bacterial genome, it became clear that *A urinae* had without doubt been detected. This is absolute proof that the blood culture isolates had not been contaminated. The germ from the valve could not have been growing, as at the time of the operation the patient was already on antibiotics. With evidence of *A urine* endocarditis in the bacterial genome, antibiotic therapy with penicillin G commenced, and the previous therapy with gentamicin continued. On the second postoperative day, a large quantity of blood was suddenly discharged through the thoracic drainage, which rapidly led to circulatory collapse. After an unsuccessful attempt at cardiopulmonary resuscitation, the patient died. Out of respect for the wishes of relatives, no autopsy was carried out.

## Summary of the Review of the Literature

From 1991 to 2017, 29 cases of *A urinae*-induced endocarditis were described in PubMed and Medline (see Table 3). One or 2 new cases are published annually. *A urinae* is a gram-positive coccus that grows in pairs and clusters, producing α-hemolysis in blood agar. It is seldom identified as a pathogen, probably due to the difficulty of isolating it microbiologically.^13^ *A urinae* has been described as causing urinary tract infections, particularly in elderly men with both local and systemic predisposing factors such as diabetes mellitus, ischemic heart disease, tumors, ureteral and urethral obstruction such as phimosis, kidney stones, prostate hyperplasia, and urological catheter carriers.^8,14^ In two thirds of these cases, a urinary tract infection and endocarditis were diagnosed simultaneously. Ninety percent of these patients had at least one of the systemic or local risk factors mentioned above.

**Table 3.** Summary of Reported Cases of *Aerococcus urinae* Endocarditis in the Literature^a^.

Source	Age/Sex	Predisposing Factors	Diagnosis	Course	Antibiotic Therapy	Outcome
Westmoreland et al^15^	49/Male	None	BC	CF and RF	β-lactam + AG	Not given
de Jong et al^8^	81/Male	BPH	BC, VS, PCR	MVR	β-lactam + AG	Alive
de Jong et al^8^	78/Male	IHD, PUC, NSCLC	BC, VS, PCR	Sepsis	β-lactam	Died
de Jong et al^8^	87/Male	CF, history of lower urinary tract symptoms	BC, VS	Severe MV regurgitation, CF	Not available	Died
de Jong et al^8^	78/Female	Ureteral stent implantation; recurring UTI	BC	Septic shock	Cefuroxime, vancomycin	Alive
Alozie et al^16^	68/Male	Urinary bladder neck sclerosis, PUC	PCR of AV	Emb (cerebral), AVR	β-lactam + AG, vancomycin	Alive
Kass et al^17^	77/Male	BPH, IHD	BC	RF, sepsis with MODS	β-lactam, vancomycin	Died
Skov et al^18^	81/Male	Aortic stenosis, UTI	BC	MI	β-lactam + AG	Died
Ebnöther et al^7^	75/Male	BPH, urethra stenosis, phimosis, PUC	PCR of AV	Septic emb (kidney, cerebral), AVR	β-lactam + AG, ceftriaxone	Alive
Schuur et al^19^	89/Male	TURP, PUC, degenerative MV	BC	Not specified	β-lactam + AG	Died
Tekin et al^20^	68/Male	BPH, DM	BC	Spondylodiscitis	β-lactam + AG	Alive
Zbinden et al^21^	48/Male	UTI	BC	Emb, Hemiplegia	β-lactam + AG, rifampicin	Alive
Zbinden et al^21^	79/Female	DM, AI	BC	Cerebral vascular attack	β-lactam	Alive
Christensen et al^22^	81/Male	Prostate-Ca, IHD, UTI	BC, UC	Emb, MI	β-lactam + AG, glycopeptide	Died
Christensen et al^22^	73/Male	TURP, BPH	BC	Emb, hemiplegia	β-lactam + AG	Died
Christensen et al^5^	78/Male	Kidney stones	BC	Uneventful	Not available	Alive
Christensen et al^5^	55/Female	DM	BC	CF	β-lactam + AG, metronidazole	Died
Christensen et al^23^	78/Male	IHD	BC, UC	RF, MI	β-lactam + AG	Died
Gritsch et al^24^	43/Male	UTI	BC	Emb, septic myocarditis	β-lactam + AG glycopeptide	Died
Slany et al^12^	69/Male	None	PCR of AV	AVR	β-Lactam + AG, ceftriaxone	Alive
Dysangco et al^25^	51/Male	UTI	BC	Uneventful	β-lactam + AG	Alive
Dysangco et al^25^	24/Male	None	BC	MVR	AG, ceftriaxone	Alive
Georgescu et al^26^	54/Female	None	BC, PCR of AV + MV	Severe sepsis, DIC	β-lactam + AG	Died
Siddiqui et al^27^	54/Male	UTI, DM, urethral strictures	BC	RF	β-lactam + AG, vancomycin	Died
Creed et al^28^	75/Male	DM, chronic kidney disease	BC	Emb (cerebral)	Ceftriaxone	Alive
Allegre et al^29^	79/Female	None	BC	AVR	Not available	Alive
Melnick et al^30^	>65/Male	UTI	BC	Emb (cerebral), MVR	β-lactam + AG	Died
Miyazato et al^31^	80/Female	UTI, renal calculi	PCR of AV	CF, AVR	Not available	Alive
Kotkar et al^32^	54/Male	Phimosis	BK, MV culture	MI, Emb. (splenic, lungs, coronal), MVR	Ampicillin	Alive
Cabezas^33^	33/Female	Bicuspid AV	BC	AVR	β-lactam + AG	Alive
Present case (2017)	49/Male	Phimosis, catheter, UTI	BC, PCR of MV	Emb (cerebral, coronal), RF, MVR	β-lactam + AG	Died

Abbreviations: BC, blood culture; CF, cardiac failure; RF, renal failure; AG, aminoglycoside; BPH, benign prostatic hyperplasia; VS, Vitek system; PCR, polymerase chain reaction; MVR, mitral valve replacement; IHD, ischemic heart disease; PUC, permanent urinary catheter; NSCLC, non–small cell lung carcinoma; MV, mitral valve; UTI, urinary tract infection; AV, aortic valve; Emb, embolization; AVR, aortic valve replacement; MODS, multiple organ dysfunction syndrome; MI, myocardial infarct; TURP, transurethral resection of the prostate; DM, diabetes mellitus; AI, aortic insufficiency; UC, urine culture; DIC, disseminated intravascular coagulation.

a A systematic Medline and PubMed review was conducted covering the period January 1, 1991, to November 30, 2017. The search terms used were “endocarditis” and “*Aerococcus urinae*.”

The average age of these patients was 66.7 years. Two thirds (20/29) of them were more than 65 years old. The youngest patient was 24 years old, and the oldest patient was 89 years old. There were 7 females and 23 males, and one was not identified. The approximate gender ratio was 1:3 (female–male). In 22 cases (approximately 70%), the antibiotic therapy comprised a combination of β-lactam antibiotics (penicillin G or flucloxacillin) and aminoglycosides (gentamicin). In some cases, vancomycin was combined with cefuroxime or a broad-spectrum antibiotic, such as piperacillin/tazobactam, was prescribed. Almost half (14 of 29) of the subjects evolved poorly, and died within the first week of hospital admission. Ten patients underwent either mitral valve replacement or aortic valve replacement. In 6 cases, the course of the disease involved septicemia, and in approximately 40% (12/29), arterial embolization, most frequently cerebral, was diagnosed. In one third of the cases, either heart failure or renal failure or both were diagnosed. In only 3 cases was the course of the disease uneventful.

## Discussion

*Aerococcus urinae* is a gram-positive bacterium. Generally considered to be of low pathogenicity, this microorganism may cause severe bloodstream infections, including phlegmon, septicemia, urosepsis, and endocarditis.^2,7,13^ Old age and underlying urologic conditions are the best-known risk factors for infection.^2,7,8^ The incidence of *A urinae* urinary tract and bloodstream infections is estimated to be 54 and 3 per 1 000 000, respectively, per year.^8^ Identifying *A urinae* can be challenging. Physicians and microbiologists should consider establishing whether endocarditis is present in patients when *A urinae* is isolated in blood. In cases of negative blood cultures, the investigation and determination of 16S rDNA PCR directly in blood culture may reveal *A urinae*.^12^ Moreover, it is possible to determine 16 rDNA PCR in the heart valve after valve-replacement surgery. The results obtained from these methods directly confirm the diagnosis of *A urinae* endocarditis. A study of 16 cases of aerococcal IE^3^ found that all the patients had received combination therapy. The potential toxicity of combination therapy requires consideration, especially in cases of therapy with aminoglycosides for elderly patients. For urosepsis, ampicillin is an attractive treatment option. Cephalosporins clearly have worse pharmacodynamics and are not suitable for treatment of *A urinae*-related IE.^3^ Alternatively, carbapenems may be considered, unless they have an unnecessarily broad spectrum.^3^

In most cases, the embolization caused by *A urinae* occurs in the central nervous system; however, in their case report, Kotkar et al describe the embolization to the right coronary artery complicated by an acute myocardium infarct. The course of *A urinae* endocarditis is not always the same. Usually, it combines with the clinical incidence of a cerebral infarct—hemiplegia,^16,22,28,30^ or transient ischemic attack.^21^ In other cases, sepsis with multiple organ failure^7,8,17,24,26^ is often associated with endocarditis generally, and is the predominant reason for the lethal course of disease. In some cases, the course of *A urinae* endocarditis develops as an expression of heart failure due to mitral valve regurgitation or aortic valve regurgitation or stenosis.^5,15,31^ Only in rare cases is the course of the disease uneventful or less noticeable.^20,25^

Although in most cases of *A urinae* endocarditis the patients are more than 65 years of age, in 2010 Dysangco et al^25^ published 2 cases in which the patients were 24 and 51 years old. Moreover, both of these present an urologic abnormality that is usual in most cases. Since the first case of *A urinae* endocarditis was published in 1991, 11 surgical procedures (including our case), 5 mitral valve replacements,^8,25,30,32^ and 6 aortic valve replacements^7,12,16,29,31,33^ have been performed. All of these were performed during this millennium. This is perhaps the result of a better understanding of the rapid course of endocarditis generally, and of the poor outcomes that occur without operative treatment of the affected heart valve. The high mortality rate may be an artefact of the low number of published cases, especially if such cases are only published if the course of the disease is unexpected or results in the sudden death of the patient. Cases of *A urinae* endocarditis that are uneventful do not arouse the interest of doctors and are not published or discussed in public. Thus, to ascertain whether the high mortality rate is actually associated with *A urinae*, one should consider a greater number of cases statistically as the current 30 cases are too few to generate summary conclusions.

In the first literature review in 2002, Ebnöther et al^7^ stated that the embolization rate with *A urinae* is somewhat higher (55%, or 6/11) than with endocarditis caused by other pathogens. In comparison, in 2017 the rate stood at 40% (12/29). Once again it is evident that with rising published case reports a declining mortality rate is expected.

Skov et al^13^ presented us in his comprehensive study of the antimicrobial susceptibility of *A urinae* to 14 antibiotics with an adequate view of the treatment options. In this study, 56 clinical isolates of *A urinae* were investigated. The following determinants were selected: minimal inhibitory concentration and time-kill curves. The results showed that a combination of only penicillin and vancomycin alone had merely a slight bactericidal effect on *A urinae*. On the other hand, instituting a treatment that included gentamicin and either penicillin or vancomycin resulted in rapid bactericidal activity.

**Figure 4.**
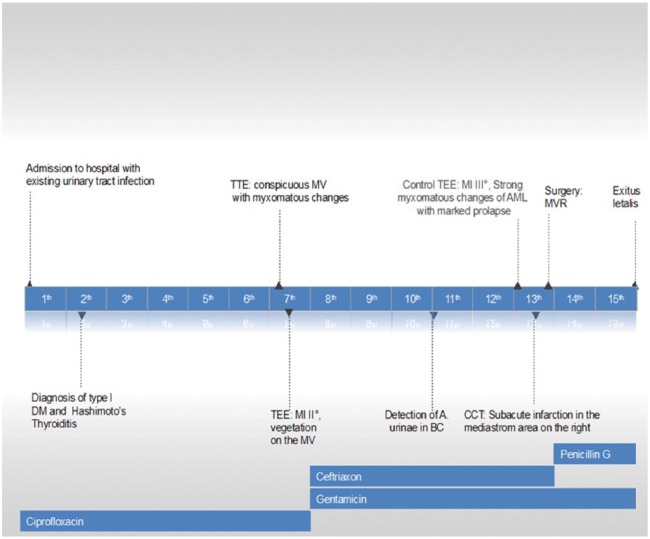
The course of the disease and therapy.

Our case report is an example of the disease that initially goes with nonspecific symptom, such as reduced general condition and signs of the urinary tract infection, continues with the course of infectious endocarditis with an undulating fever, positive blood culture for the bacterium such as damaged mitral valve, and finally leads to a poor outcome (see Figure 4). A retrospective analysis of the case teaches us that there is still an uncertainty of choosing the proper operation time. Current criteria are based on the size of vegetation, valve insufficiency grade, and appearance of the complications such as heart failure or embolic events. The search for further criteria would be meaningful. The establishment of the 16S rDNA PCR in daily use has extended the availability of the microbiological diagnostic, especially to find the specific pathogens of the disease. However, not all health care facilities can afford the equipment needed for genomic bacterial testing. In case of infectious disease and negative blood cultures, the patient should be moved to the center with the necessary diagnostic option. On the other hand, there should be an opportunity to take an immediate option of surgical treatment of the damaged heart valve.

## Conclusion

Generally considered to be of low pathogenicity, this microorganism may cause severe bloodstream infections, including endocarditis. *A urinae* endocarditis is a rare disease that mostly affects elderly men with urinary tract pathologies and comorbidities. The course of the disease is severe and often has a lethal outcome. Identifying *A urinae* can be challenging. Physicians and microbiologists should consider establishing whether endocarditis is present in patients when *A urinae* is isolated in blood. A 16S rDNA PCR investigation of bacterial genome detection gives a secure proof of the presence of *A urinae*. It provides firm evidence that the blood culture isolates had not been contaminated. Genome detection is indicated especially in cases where the results of the blood culture are negative. Because of the high risk of embolism, rapid treatment should focus on the diseased heart valve. Based on existing data and the experience gained from handling cases, treatment with β-lactam and aminoglycosides is recommended. It is recommended that operative therapy take place as soon as possible.
